# Very Late Iliac Venous Stent Migration Into the Right Ventricle With Tricuspid Valve Injury Five Years After Implantation: A Case Report

**DOI:** 10.7759/cureus.96277

**Published:** 2025-11-07

**Authors:** Salma El Manir, Emane Phalex, Fouad Nya, Hatim Yousfi, Younes Moutakiallah

**Affiliations:** 1 Cardiac Surgery, Mohammed V Military Training Hospital, Rabat, MAR

**Keywords:** cardiac surgery, endovascular complications, iliac vein stent, stent migration, tricuspid valve injury

## Abstract

Stent migration into the right heart chambers is an uncommon but potentially life-threatening complication of venous stenting. Most reported cases occur shortly after implantation. A 72-year-old man with prior left iliac vein stenting presented five years later with dyspnea and hemoptysis. Imaging revealed migration of the iliac stent into the right ventricle, in direct contact with the tricuspid valve apparatus. The patient underwent successful surgical removal on a beating heart, including neocordae implantation and annuloplasty ring placement. Postoperative recovery was uneventful. This case illustrates the potential for very late stent migration, emphasizes the risk of valvular damage, and underlines the importance of long-term surveillance following venous stent implantation.

## Introduction

Venous stenting is being performed with increasing frequency for the management of chronic venous disease, particularly in patients with post-thrombotic syndrome, and is generally associated with favorable outcomes [1,2]. Despite its effectiveness, this intervention is not without risk. Early complications such as stent thrombosis or migration have been well documented and are usually observed within weeks or months after implantation [3].

By contrast, delayed complications are uncommon but may carry significant morbidity. The overall incidence of stent migration in venous interventions has been estimated at less than 3% in large endovascular series [4]. Among these, very late migration into the cardiac chambers is exceptionally rare. When it occurs, intracardiac migration can lead to serious sequelae, including arrhythmias, valvular injury, right heart dysfunction, or embolic events. Most cases are reported within months of implantation; very delayed migration, occurring several years later, is exceptional.

Here, we present a rare case of iliac venous stent migration into the right ventricle five years after implantation, complicated by entrapment in the tricuspid valve and structural damage requiring surgical extraction and valve repair.

## Case presentation

The patient was a 72-year-old man, a former smoker (50 pack-years, quit 16 years earlier), with android obesity (body mass index of 37 kg/m²). He had undergone venous stenting five years earlier (February 2020) for post-thrombotic syndrome secondary to extensive iliofemoral thrombosis. A nitinol EPIC™ stent (Boston Scientific; 8 × 80 mm) had been placed in the left common iliac vein following balloon angioplasty, with restoration of venous patency.

**Figure 1:**
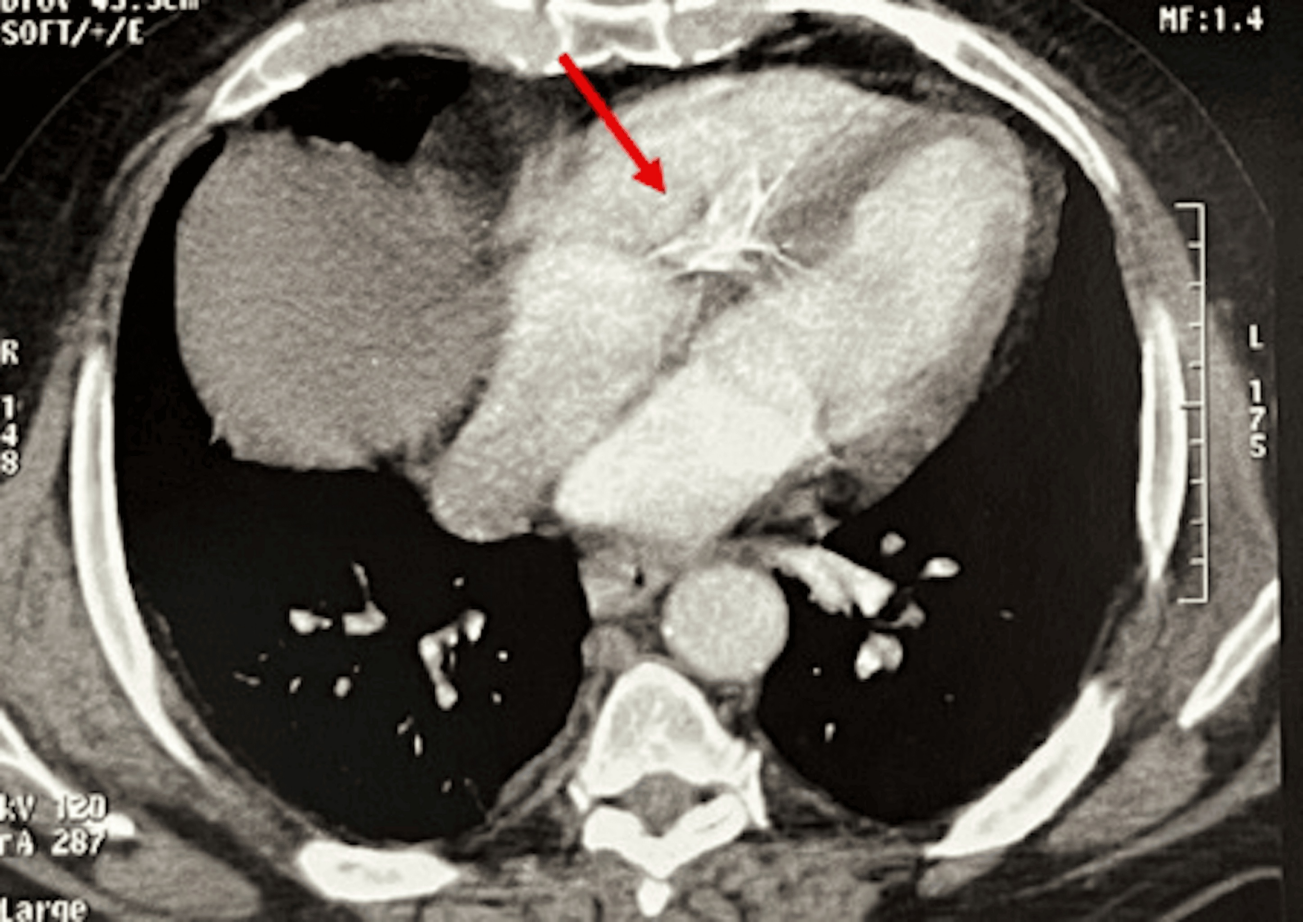
Contrast-enhanced axial CT image demonstrating a metallic structure (red arrow) lodged within the right atrium and right ventricle, consistent with a migrated iliac venous stent.

**Figure 2:**
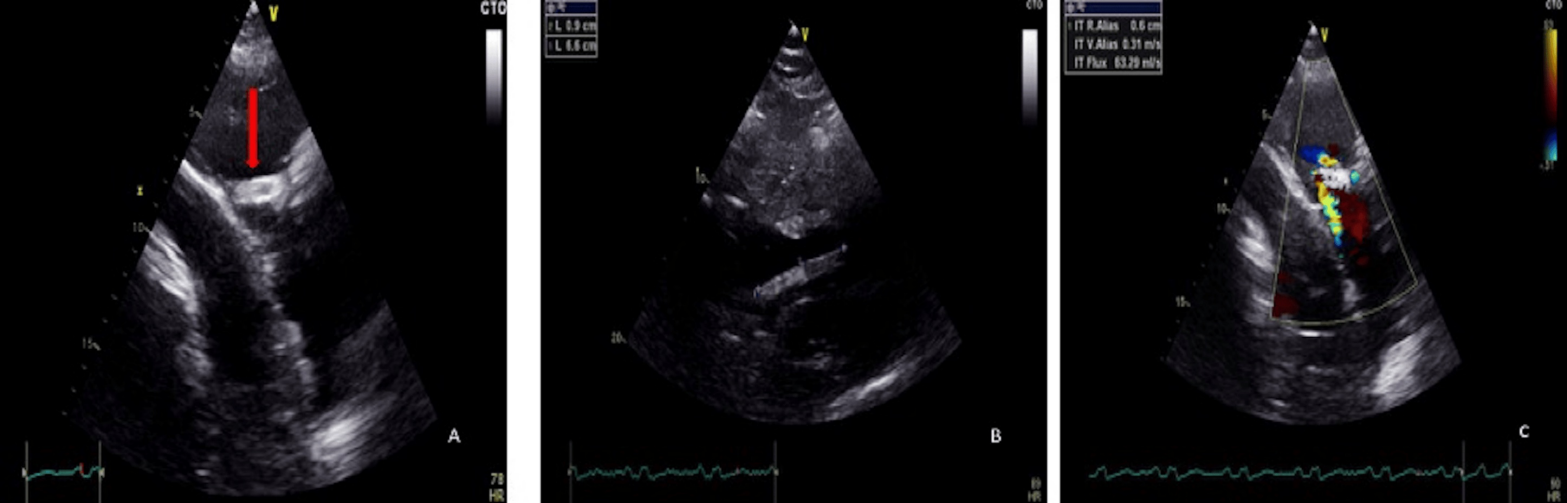
Transthoracic echocardiographic views. (A) Parasternal long-axis view showing a linear echogenic structure (red arrow) extending into the right ventricle, compatible with the venous stent. (B) Apical four-chamber view highlighting the stent embedded near the tricuspid subvalvular apparatus. (C) Color Doppler view revealing mild tricuspid regurgitation.

Five years after iliac vein stenting, in April 2025, the patient presented with hemoptysis, exertional dyspnea (New York Heart Association Class III), orthopnea, and bilateral leg edema, without prior cardiac history. A contrast-enhanced chest CT scan incidentally revealed a metallic foreign body within the right heart (Figure 1). Transthoracic echocardiography showed an 80 × 10 mm metallic structure extending from the right atrium into the right ventricle, in contact with the tricuspid subvalvular apparatus (Figure 2).

**Figure 3:**
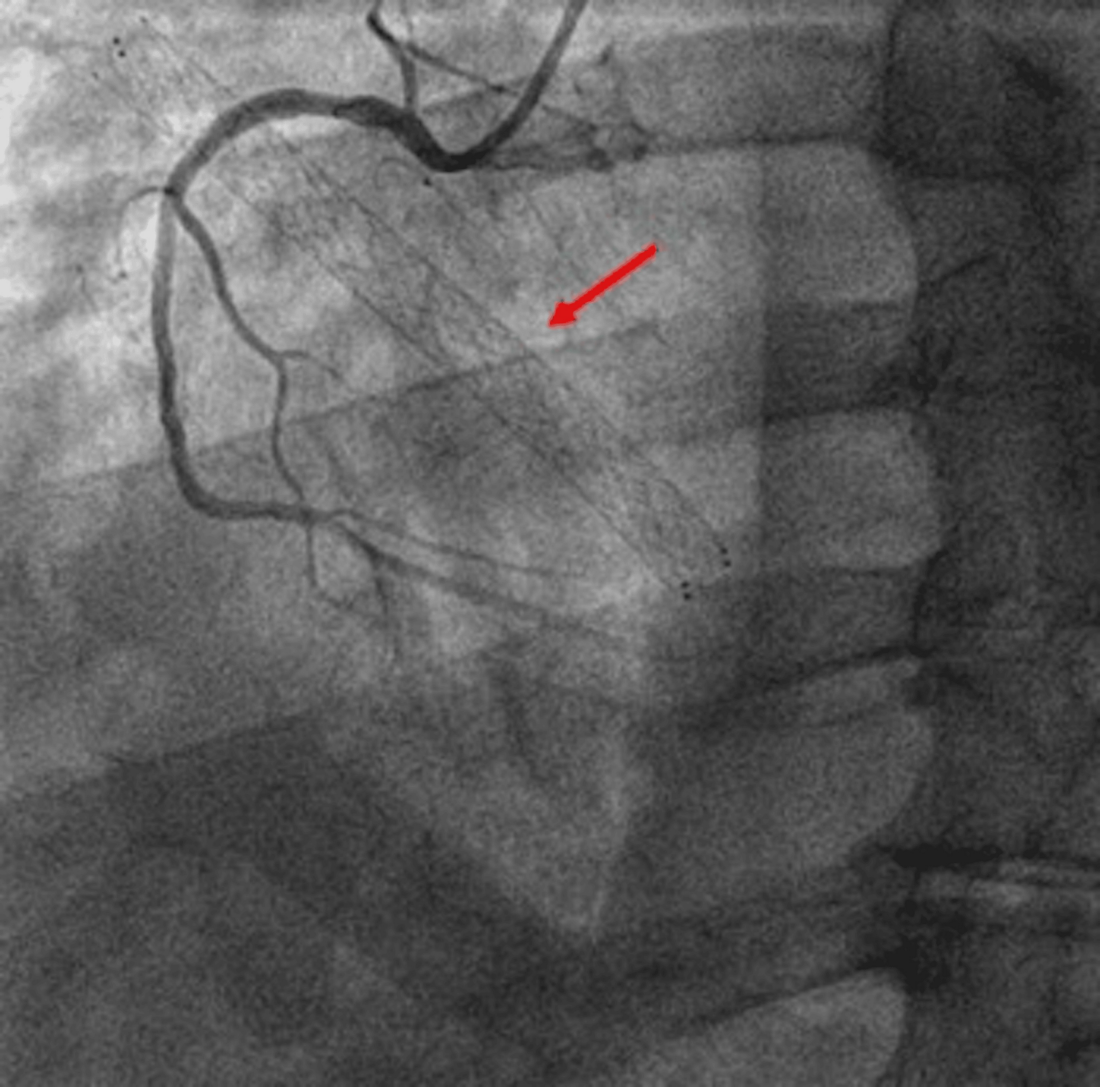
Coronary angiography demonstrating the migrated iliac venous stent as a radiopaque linear structure (red arrow) within the right ventricle, located near the tricuspid valve and the right coronary artery.

Minimal tricuspid regurgitation was noted, with preserved biventricular function and pulmonary artery systolic pressure of 28 mmHg. Despite only mild regurgitation, symptoms of right heart failure were attributed to mechanical interference of the migrated stent. There was no evidence of endocarditis or thrombus formation. Coronary angiography excluded significant coronary disease and confirmed the stent’s proximity to the right coronary artery (Figure 3).

The patient was referred for surgical extraction of the migrated stent. Beating-heart removal was performed via median sternotomy under cardiopulmonary bypass. The superior vena cava was cannulated first, followed by right atriotomy. Under direct visual guidance, the inferior venous cannula was then inserted into the inferior vena cava to ensure safe and controlled venous drainage.

The iliac stent was identified as a single continuous metallic structure extending from the right atrium into the right ventricle, firmly adherent to the septal leaflet of the tricuspid valve, which was tethered to the interventricular septum.

**Figure 4:**
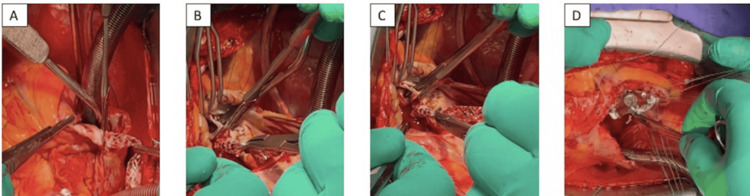
Intraoperative images illustrating surgical management of the migrated iliac venous stent. (A) Careful extraction of the proximal portion of the stent from the right atrium. (B) Delicate mobilization and removal of the stent from the tricuspid subvalvular apparatus. (C) Complete extraction of the metallic stent under direct vision. (D) Tricuspid valve repair using an annuloplasty ring.

Because of its length and firm adherence to intracardiac structures, the stent was sectioned intraoperatively to facilitate extraction. The atrial portion, which was adherent to the endocardium of the right atrium, was removed first with meticulous dissection (Figure 4A). The remaining ventricular portion was intimately embedded in the subvalvular apparatus and firmly adherent to the chordae tendineae of the anterior leaflet of the tricuspid valve. Complete removal required partial resection of the involved chordae (Figures 4B, 4C). Tricuspid valve repair was then performed using Gore-Tex® neochordae implantation and placement of a 30 mm Medtronic Contour 3D® annuloplasty ring (Figure 4D), restoring satisfactory valvular function.

Postoperative recovery was uneventful. The patient was extubated after six hours, remained in intensive care for 24 hours, and was discharged on postoperative day 14. Follow-up transthoracic echocardiography demonstrated preserved right ventricular function with only trace tricuspid regurgitation.

## Discussion

Venous stent migration into the cardiac chambers is a rare but potentially life-threatening complication of endovascular therapy. Most reported cases occur within weeks or months after implantation, typically due to technical factors such as stent undersizing, inadequate wall apposition, or high venous flow dislodging the device [1]. In contrast, delayed migration occurring several years later, as in our case, is exceptional and likely multifactorial.

Possible mechanisms for late migration include gradual mechanical fatigue of the stent, chronic venous wall remodeling, or progressive loss of adherence due to evolving pressure gradients [2]. In this patient, obesity and increased intra-abdominal pressure may have contributed to slow loosening over time, eventually resulting in embolization into the right heart.

The overall incidence of stent migration in venous interventions is low (<3%) [1,2]. However, late intracardiac migration with valvular involvement is exceedingly uncommon, with only a few published cases describing tricuspid injury or right-sided heart dysfunction [3,4]. What distinguishes our case is the five-year latency between stent implantation and presentation, as well as the direct mechanical damage to the tricuspid valve that required complex surgical repair. Similar reports have rarely involved neochordae reconstruction and annuloplasty [5,6].

Percutaneous retrieval has been described for freely mobile stents detected early, before valvular embedding [7]. In contrast, when structural entrapment or leaflet injury is present, surgical extraction with valve repair remains the only feasible approach [8]. Beating-heart surgery was chosen in our patient to preserve myocardial function and allow continuous assessment of right ventricular performance during manipulation.

This case underscores the importance of long-term imaging surveillance after venous stenting, particularly in patients with altered venous anatomy or increased intra-abdominal pressure. Although no standardized follow-up protocol currently exists, annual duplex ultrasonography or cross-sectional imaging may facilitate early detection of stent displacement and help prevent severe complications. Reporting such rare events increases awareness and contributes to the development of preventive strategies and surgical management guidelines for venous stent migration.

## Conclusions

This case illustrates an exceptionally rare instance of very late intracardiac migration of an iliac venous stent with tricuspid valve involvement. It highlights that stent embolization can occur even several years after implantation, underscoring the need for lifelong vigilance and structured imaging follow-up in patients with venous stents. Early recognition through echocardiography or CT is crucial to prevent irreversible valvular injury and right-sided heart dysfunction. When migration results in valvular entrapment, prompt surgical extraction with valve repair provides a safe and definitive solution. Reporting such uncommon events raises clinical awareness, informs long-term surveillance strategies, and refines the surgical management of venous stent complications.
